# Recurrent ascending cholangitis secondary to food reflux following transduodenal resection of an ampullary adenoma

**DOI:** 10.1308/rcsann.2024.0110

**Published:** 2025-03-25

**Authors:** M Fouad, MW James, AM Zaitoun, M Hanks, DN Lobo

**Affiliations:** ^1^ University of Nottingham, UK; ^2^ Nottingham University Hospitals NHS Trust, UK; ^3^ University of Pennsylvania, Philadelphia, USA

**Keywords:** Ampulla of Vater, Transduodenal ampullectomy, Recurrent cholangitis, Biliary lithiasis, Food reflux

## Abstract

Ampullary lesions, including adenomas and early-stage carcinomas, pose a diagnostic and therapeutic challenge because of their location and proximity to the pancreatic and bile ducts. Transduodenal ampullectomy offers a targeted approach for the resection of these lesions while preserving the integrity of the pancreaticobiliary system. Moreover, transduodenal ampullectomy is associated with favourable postoperative outcomes, including low rates of morbidity and mortality, as well as preservation of pancreatic and biliary function. However, potential complications such as ascending cholangitis pancreatic leakage, bleeding and duodenal stenosis can occur, which would impact the postoperative quality of life. Addressing these outcomes might require either endoscopic procedures or surgical interventions. We present an exceedingly uncommon case of recurrent ascending cholangitis resulting from reflux of food particles into the common bile duct that was treated successfully with a Roux-en-Y hepaticojejunostomy and gastroenterostomy.

## Background

Transduodenal ampullectomy (TDA) is a well-described procedure for ampullary tumours.^1–5^ However, ampullectomy may lead to postoperative complications, including early sequelae such as pancreatitis, bleeding, perforation and cholangitis, which can often be adequately managed by nonoperative treatment. Late complications, such as pancreatic or biliary stenosis (0%–8%), can be addressed with endoscopic sphincterotomy, stent placement and balloon dilation.^6^ Reflux of food particles into the common bile duct, although relatively common after choledochoduodenostomy, has not been previously reported following TDA.^7^ We report a rare case of recurrent ascending cholangitis arising from the reflux of food particles into the common bile duct following surgical TDA.

## Case history

A 68-year-old woman had undergone an open cholecystectomy and TDA for a tubulovillous adenoma of the ampulla of Vater in 2011. Three years later, she experienced recurrent episodes of jaundice and cholangitis necessitating frequent endoscopic retrograde cholangiopancreatography (ERCP) to clear the common bile duct (CBD). Initially, it was believed that the recurrent cholangitis was caused by the formation of primary CBD stones and cholestasis, leading to the patient being placed on regular ursodeoxycholic acid to reduce recurrence.

**Figure 1:**
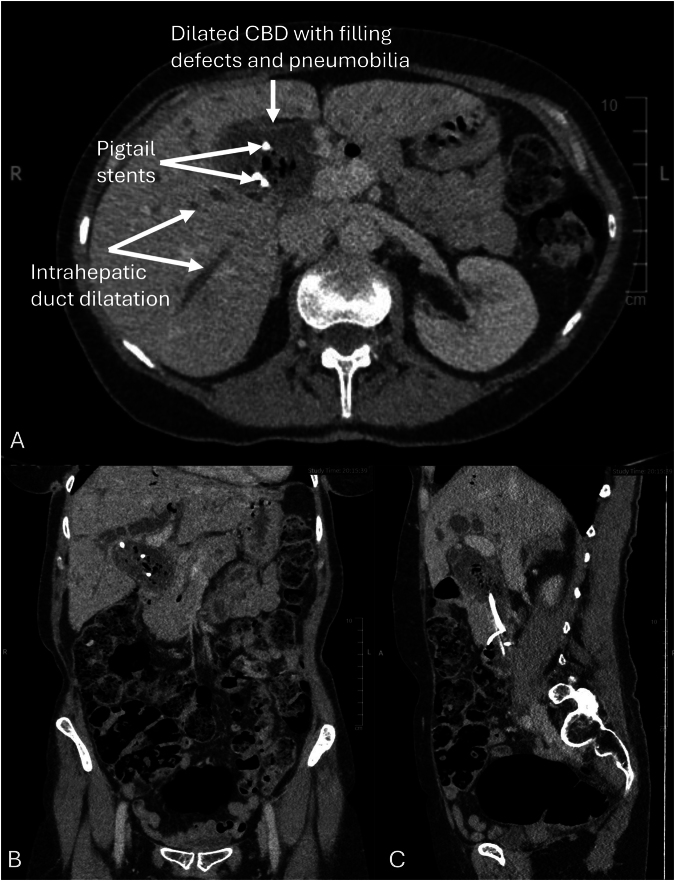
Axial (A), coronal (B) and sagittal (C) computed tomography images showing dilated intra- and extrahepatic bile ducts, pneumobilia, and multiple filling defects in the common bile duct (CBD) next to the pigtail stents.

**Figure 2:**
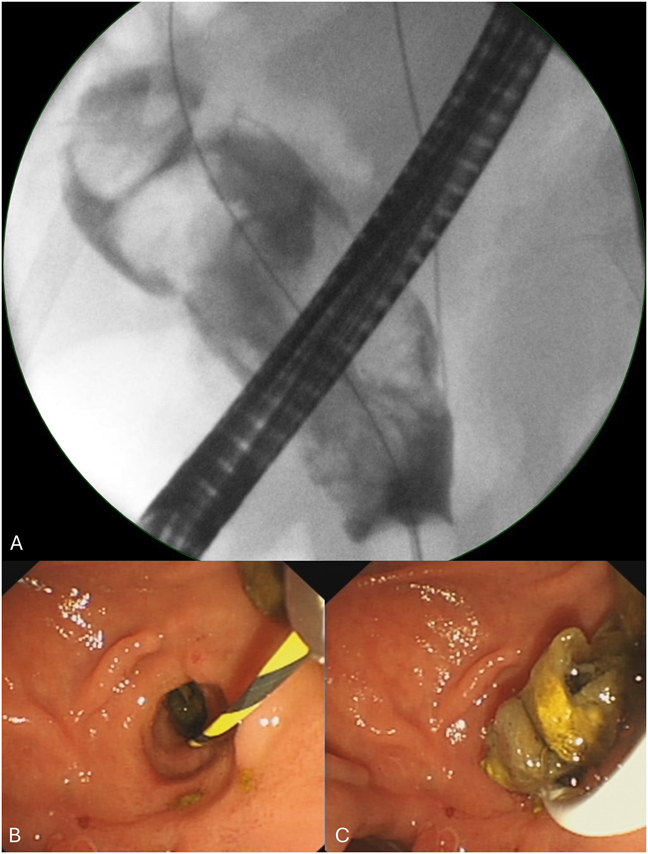
Fluoroscopy at endoscopic retrograde cholangiopancreatography showing a grossly dilated common bile duct with multiple filling defects within it (A). The transduodenal ampullectomy site was wide open (B) and food debris were extracted with a 15–18mm balloon catheter (C).

However, further episodes of cholangitis continued to occur. She went on to have a total of 13 ERCPs for duct clearance between 2014 and December 2023. When she developed recurrent cholangitis two weeks after an ERCP in 2024, further imaging studies including computed tomography (CT) scanning (Figure 1) and magnetic resonance cholangiopancreatography (MRCP) were performed. CT and MRCP showed dilated intra- and extrahepatic bile ducts, pneumobilia and filling defects in the CBD next to the stent. Subsequent ERCP revealed the presence of food particles rather than stones within a hugely dilated extrahepatic biliary tree (Figure 2). Further CBD clearance and stent placement were performed as a bridging intervention until definitive surgical management.

Following multidisciplinary discussion, a decision was made to proceed with open hepaticojejunostomy for bile duct reconstruction and gastrojejunostomy. The rationale was to disconnect the CBD (biliary system) from the alimentary stream. Moreover, this would allow access for endoscopic ultrasound and ERCP for future surveillance of the ampullectomy bed recurrence and instrumentation of the distal CBD in case food particles recollected.

**Figure 3:**
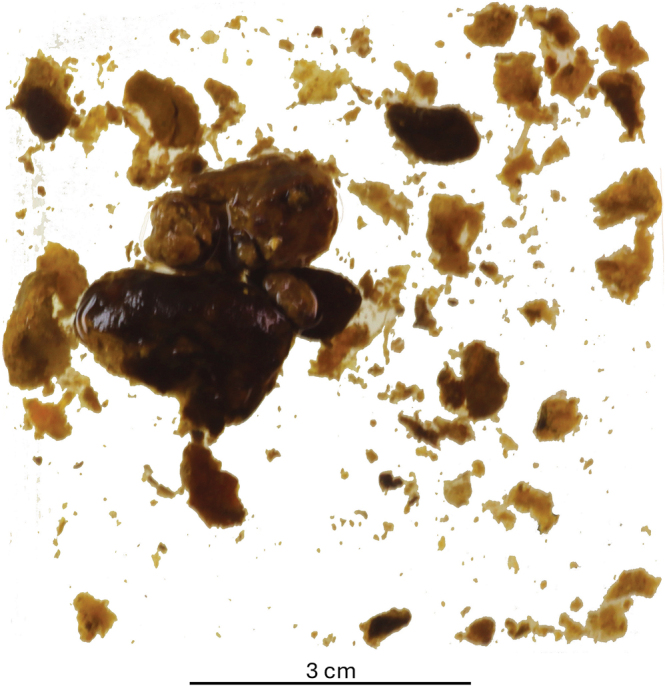
Multiple clumps of food particles and vegetable matter were extracted from the extrahepatic bile ducts at surgical exploration.

Intraoperatively, after transection of the common hepatic duct, multiple food particles and vegetable matter (Figure 3) were retrieved from the common hepatic duct and distal CBD along with the stents. This was performed under choledochoscopic guidance, with instrumentation and copious irrigation. Completion choledochoscopy confirmed ductal clearance. The distal CBD was oversewn and a Roux-en-Y hepaticojejunostomy and gastrojejunostomy were performed. A 16G trucut biopsy of the liver was also taken. The postoperative course was uneventful, and the patient was discharged on the fifth postoperative day.

**Figure 4:**
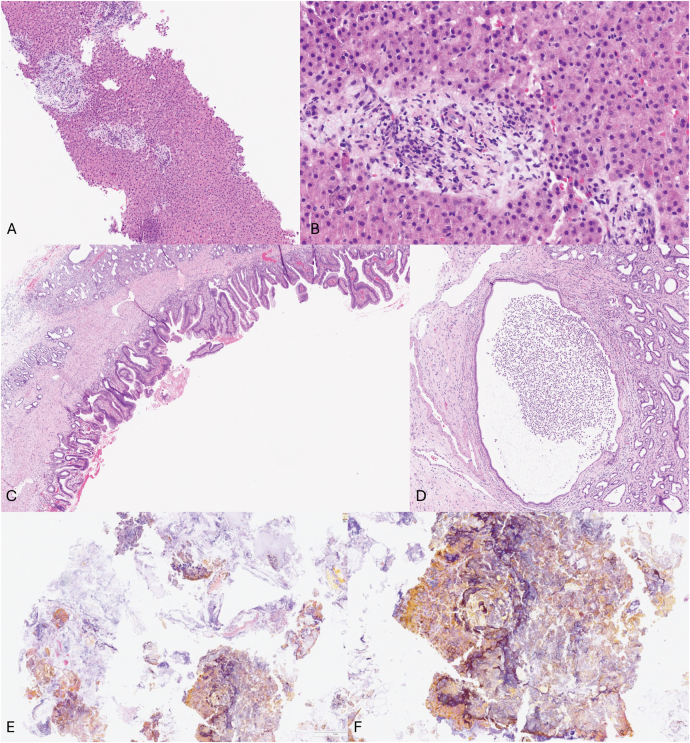
Liver biopsy: portal tract areas showed moderate mixed inflammatory cell infiltrate including predominantly polymorphs and lymphocytes (A, B). There was infiltration of the bile ducts by polymorphs with focal interface hepatitis. There was no evidence of cholestasis or steatosis. The parenchyma showed lobular inflammation. The appearances were compatible with ascending cholangitis. Section of the bile duct: sections showed bile duct epithelium with regenerative changes, ulceration and focal abscess formation (C, D). Food material: sections showed plant and food material admixed with bile (E, F). All sections were stained with haematoxylin and eosin.

Histopathological examination of the liver biopsy (Figure 4A,B) revealed evidence of ascending cholangitis and bridging fibrosis, but no evidence of steatosis or cholestasis. The bile duct showed epithelial regenerative changes with ulceration and abscess formation with no evidence of dysplasia or malignancy (Figure 4C,D). Finally, food material and plant matter mixed with bile was confirmed in the specimen from within the CBD (Figure 4E,F).

The patient remained clinically well and symptom-free when last seen 9 months postoperatively.

## Discussion

Surgical ampullectomy, also known as TDA, for ampullary lesions was initially described by Halsted in 1899.^8^ TDA has been a feasible and less-invasive alternative to pancreaticoduodenectomy. TDA has comparable outcomes with endoscopic ampullectomy for the treatment of early-stage ampullary cancer and benign tumours, and is particularly useful for high-risk surgical patients or when endoscopic ampullectomy is inadequate or not feasible.^2^ In a recent meta-analysis involving 13 TDA data sets, the pooled overall complication rate was found to be 28.3% (95% CI 19.0 to 37.7), including short-term complications (acute pancreatitis, bleeding, perforation, infection and leakage) and long-term complications (biliary stricture and cholangitis).^6^ Cholangitis has been attributed to the development of biliary sludge around non-absorbed suture material or secondary biliary stone formation.^9^ However, there was no reference to food regurgitation into the CBD as a direct aetiology for recurrent cholangitis in the literature following TDA.

Patients who were subjected to choledochoduodenostomy in the past were at risk of reflux of food particles into the CBD and subsequent cholangitis because of loss of the valvular function of the ampulla of Vater. This is different to sump syndrome, which is related cholestasis following an angled anastomosis as in choledochoduodenostomy, which is another reason for stone formation and cholangitis.^7,10^

There has been a fixed ideation that long-term cholangitis after TDA is secondary to biliary stone formation, which contrasts with the traditional teaching of spontaneous passage of newly formed CBD stones after ampullectomy or even sphincterotomy. This is corroborated by the approximately 3% rate of CBD stone recurrence following sphincterotomy via ERCP, so stone-related cholangitis should be even less common after excision of the whole ampulla.^11^ This ideation is emphasised in the current case in which the filling defects in the CBD and cholangitis were thought to be caused by recurrent stones rather than reflux of food. Reflux of food was only suspected when the patient developed further cholangitis and filling defects two weeks after duct clearance at ERCP. This was successfully treated with a Roux-en-Y hepaticojejunostomy and partial diversion of the food stream from the ampullectomy site by a gastrojejunostomy.

Recurrent cholangitis and eventually biliary cirrhosis following TDA has to be revisited and extensively investigated to exclude underlying retrograde food reflux into the CBD. Currently, there is no consensus or guidance on how to manage such cases. However, based on our case, we suggest that a Roux-en-Y hepaticojejunostomy and gastrojejunostomy would be an effective and reasonable definitive option for such patients.

## Conclusion

This case report demonstrates that flood reflux into the biliary tree can occur after TDA, and perhaps after endoscopic ampullectomy or sphincterotomy. Recurrent cholangitis and biliary filling defects should not be attributed to new primary biliary lithiasis, and food reflux must be considered. Consideration of Roux-en-Y hepaticojejunostomy and gastroenterostomy may be definitive treatment to prevent food reflux and recurrent cholangitis, while still preserving access to the ampullectomy site for secondary surveillance or treatment.

## Conflicts of interest

None of the authors has a direct conflict of interest to report. DNL has received an unrestricted educational grant from B Braun for unrelated work. He has also received speaker’s honoraria for unrelated work from Abbott, Nestlé and Corza.

## Author contributions

All authors: study design, data collection and data interpretation. MF and DNL: writing the manuscript. All authors: critical review of the manuscript and final approval. All the authors had access to the data.
